# The relationship between indices of iron status and selected anthropometric cardiovascular disease risk markers in an African population: the THUSA study

**DOI:** 10.5830/CVJA-2011-015

**Published:** 2011-10

**Authors:** OR Aderibigbe, PT Pisa, RL Mamabolo, HS Kruger, HH Vorster

**Affiliations:** Centre of Excellence for Nutrition, Faculty of Health Sciences, North-West University, Potchefstroom, South Africa; Centre of Excellence for Nutrition, Faculty of Health Sciences, North-West University, Potchefstroom, South Africa; Centre of Excellence for Nutrition, Faculty of Health Sciences, North-West University, Potchefstroom, South Africa; Centre of Excellence for Nutrition, Faculty of Health Sciences, North-West University, Potchefstroom, South Africa; Centre of Excellence for Nutrition, Faculty of Health Sciences, North-West University, Potchefstroom, South Africa

**Keywords:** iron indices, anthropometry, cardiovascular diseases, African, THUSA study

## Abstract

**Abstract:**

There is evidence that certain indices of iron status are associated with anthropometric measures, which are used independently as markers of cardiovascular disease (CVD) risk. This study examined whether this association exists in an African population. The study was a cross-sectional comparative study that examined a total of 1 854 African participants. Ferritin was positively associated with body mass index (BMI), waist circumference (WC), waist-to-hip ratio (WHR), percentage body fat and subscapular skinfold thickness. Serum ferritin concentration was higher in the high-WHR category than the normal-WHR category for both genders. Additionally, WC and WHR increased with increasing ferritin concentrations in both genders. Serum iron was lower in the obese than the normal-weight and pre-obese women only. In this population-based study, increased serum ferritin concentrations associated positively with increased WHR and WC, indicating that individuals or populations at risk of iron overload as defined by high serum ferritin concentrations may be at a greater risk of developing CVD.

## Abstract

South Africa is experiencing a health transition, associated with a triple burden of disease characterised by a high prevalence of undernutrition-related infectious diseases, the emergence of non-communicable diseases, and the human immunodeficiency virus/acquired immune deficiency syndrome (HIV/AIDS) pandemic.^1^ Micronutrient deficiencies still remain a major public health challenge in most developing countries. Iron-deficiency anaemia is the most common nutritional deficiency in the world and it has negative consequences for growth and health.^2^,^3^ The prevalence of iron deficiency is highest in developing countries and its causes are multi-factorial.^4^ In South Africa, anaemia has been reported in seven to 29% of pregnant women^5^-^7^ and 57% of pregnant teenage girls.^8^ The World Health Organisation^2^ estimated that 26.4% of non-pregnant women of reproductive age in South Africa had haemoglobin concentrations below 12 μg/dl. Charlton^9^ also reported a 13% prevalence of anaemia among an elderly South African population.

Iron is a key element in many biochemical processes and shortage of iron causes damage to cells and organs. On the other hand, excess iron could be harmful because it is able to catalyse the formation of highly reactive oxygen and hydrogen radicals when present in the unbound state.^10^ Because of the ease with which additional iron can be provided to iron-replete individuals through iron-fortified foods or iron supplements, and the limited ability to excrete the mineral, the consequences of iron excess are as relevant nutritionally as the liabilities of iron deficiency. A high prevalence of iron overload (15%) was reported among black males across sub-Saharan Africa who customarily drink a traditional fermented beverage with a high iron content.^10^ A genetic predisposition to iron overload has also been identified in Africans.^10^

Due to rapid urbanisation, lifestyle changes and the adoption of Western diets, obesity has become a growing problem in developing countries. Countries undergoing transition, such as China, Brazil and South Africa are particularly affected and have an increasing prevalence of obesity across all economic levels and age groups.^11^ In South Africa, 30% of men and 55% of women have been classified as overweight or obese.^12^

Cross-sectional studies have indicated that measures of iron status are positively associated with cardiovascular disease (CVD) risk factors, and this association is hypothesised to be mediated by adiposity.^14^,^15^ Additionally, iron deficiency has been reported to be positively associated with anthropometric indicators such as waist circumference (WC), body mass index (BMI) and waist-to-hip ratio (WHR),^13^,^14^ which are now used independently as markers for CVD risk.^15^ Establishing the relationship between measures of iron status and these anthropometric CVD risk markers may give an indication whether the iron status of a population can predict its CVD risk. In view of this, the present study was aimed at examining the relationship between measures of iron status (ferritin, serum iron and haemoglobin concentrations, total iron-binding capacity and percentage transferrin saturation) and selected anthropometric CVD risk markers in an African population.

## Methods

The THUSA (Transition and Health during Urbanisation of South Africans) study was conducted from 1996 to 1998 in the North West province of South Africa.^16^ It was a cross-sectional comparative study in which a community-based sample of 1 854 apparently healthy black volunteers (15 years and older) were recruited from 37 randomly selected sites, using a statistical model that ensured a representative sample from five levels of urbanisation: deep rural, commercial farms, informal settlements, ‘middle-class’ urban and ‘upper-class’ urban. Pregnant and lactating women, individuals taking chronic medication, those with oral temperatures above 37°C and inebriated volunteers were excluded.

Permission to conduct the study in specific areas with advice on recruitment procedures was obtained from the North West Department of Health, tribal chiefs, community leaders, headmasters of high schools and mayors. The study was approved by the Ethics Committee of the North-West University (ethics number: 4M5-95) and all participants signed an informed consent form. Participants were fasted (10–12 hours) for baseline blood sampling and other measurements. They received lunch after completion of the tests. All participants received feedback regarding their blood pressure, fasting glucose concentrations and haemoglobin values. Where necessary, participants were referred to their nearest health facility for further diagnosis and treatment. Travelling expenses of participants were covered.

Questionnaires were designed for this study population and were validated using appropriate methods.^16^ The questionnaires were administered during individual interviews conducted by the researchers and specially trained African field workers in the language of the participant’s choice.

The demographic questionnaire included questions on type of housing, access to electricity, water source, sanitation, personal and household income, health history (also of close family members), number and ages of people living in the same house, ownership of property, education level as well as smoking and drinking habits.

Anthropometric measurements were done in triplicate by postgraduate biokinetics students and standardised by a level III anthropometrist. Height was measured to the nearest 0.5 cm with a stadiometer (Invicta, IP 1465, UK) and weight was determined on a portable electronic scale to the nearest 0.1 kg (Precision Health Scale, A & D Company, Japan) with the participants in light clothing. Skinfold thickness and body circumferences of participants in their underwear were measured with calibrated instruments (Holtain® unstretchable metal tape; John Bull® calipers). All the measurements were done according to standards of the International Society for the Advancement of Kinanthropometry (ISAK).^17^ BMI was calculated by dividing weight in kilograms by height in metres squared. Waist-to-hip ratio was calculated by dividing WC by hip circumference. Percentage body fat was calculated using Siri’s equation: (4.95/density – 4.50) × 100.^18^ Total body density was derived from Durnin and Womersley’s equation,^19^ using the sum of biceps, triceps, subscaplar and supra-iliac skinfold thicknesses.

Two nurses examined the participants for clinical signs of malnutrition. Oral temperatures were taken and blood pressure was recorded in triplicate using a sphygmomanometer (Tycos®) with adjustable cuffs of different sizes.

HIV status was determined anonymously with an enzymeimmunological method (enzymum-Test®, anti-HIV 1 and 2 and subtype Ø, Boehringer Mannheim, Germany, cat no 1557319).

Blood was drawn from the vena cephalica using a sterile butterfly infusion set (Johnson & Johnson, 21G, 19 mm) and syringes. For preparation of serum, 5 ml blood was allowed to clot in glass tubes, centrifuged at 3 000 rpm for 15 minutes (Universal 16R™, Hettich, with cooling facilities) and transferred into storage tubes. Citrated blood was prepared by drawing 4.5 ml blood into a syringe containing 0.5 ml 1 mol/l citrate (pH 4.5–4.8). These samples were centrifuged for 10 minutes at 3 000 rpm in plastic siliconised tubes and the plasma was stored in tubes. All serum and plasma samples were immediately stored at –18°C to –20°C in the field for two to four days, and afterwards at –84°C in the laboratory.

All laboratory analyses were done within a year of blood collection. Haemoglobin concentration was measured in the field on EDTA (ethylenediaminetetraacetic acid) blood. Serum ferritin concentration was measured using an immunoradioactive assay (Ferritin MAb Solid Phase Component System; Benton Dickson & Co, Orangeburg, NY) with an Auto Gamma 500C counting system (United Technologies, Packard, III). Serum iron concentration and total iron-binding capacity (TIBC) were determined spectrophotometrically with an RA-1000 automated system (Technicons, Tarrytown, NY) using a colorimetric method (Fe SYS 1 and Test-Combination Iron-binding Capacity; Boehringer Mannheim). Percentage transferrin saturation was calculated by finding the molar ratio of serum iron and twice the serum transferrin using the formula: percentage transferrin saturation = [serum iron (μg/dl) × 100/transferrin (mg/dl) × 2].

## Statistical analysis

Data were analysed using SPSS (Statistical Package for Social Sciences) version 17 and presented as geometric means (G-means) and standard error of means (SE). The minimum and maximum values were also stated. Spearman correlation was used to assess the relationship between iron indices and anthropometric indicators. Partial correlations after adjusting for age, BMI and smoking were further assessed. A stepwise regression method was used to identify valid confounders in this particular population. Age, BMI and smoking were treated as confounders. HIV status did not modify or confound the iron status parameters, so it was not adjusted for in the analysis.

To further assess the relationship between iron status and anthropometric indicators, men and women were grouped and analysed in different WHR and BMI categories.^20^ Additionally, men and women were grouped and analysed in three ferritin groups: (1) low-ferritin group (serum ferritin concentration below 12 μg/l), (2) normal-ferritin group (serum ferritin concentration between 12 and 150 μg/l) and (3) high-ferritin group (serum ferritin concentration above 150 μg/l). These cut-off points are the clinical cut-off points recommended by standard dietetic practice.^21^ Multivariate analysis was used to assess significant differences between different groups before and after adjusting for age, BMI and smoking. Statistical significance was set at *p* < 0.05.

## Results

Table 1 outlines the anthropometric and iron indices of the participants. All iron indices were better in men than women (*p* < 0.0001) before and after adjusting for age, BMI and smoking. Women had a higher mean (geometric) BMI (*p* < 0.0001) and percentage body fat (*p* < 0.0001) than men. Waist circumference *p* = 0.006) and WHR (*p* < 0.0001) were significantly higher in men than women before adjusting for age, BMI and smoking. Age did not differ significantly (*p* = 0.076) between men and women.

**Table 1:** Anthropometric And Haematological Characteristics Of Participants

	*Men (n = 711, 42.80%)*		*Optimum cut-off point*	*Women (n = 952, 57.20%)*		*Optimum cut-off point*
*Variable*	*G-Mean (SE)*	*Min, max*		*G-Mean (SE)*	*Min, max*	
Age (years)	34.32 (0.58)	15.00, 82.00	–	35.04 (0.46)	15.00, 90.00	–
Serum Fe (μmol/l)	16.52 (0.33)	0.74, 73.42	≥ 11^22^	13.13^*#^ (0.24)	0.26, 59.85	≥ 11^22^
Serum TIBC (μmol/l)	63.86 (0.49)	28.77, 197.22	≤ 73^22^	68.21^*#^ (0.44)	29.13, 171.12	≤ 73^22^
Transferrin saturation (%)	25.86 (0.53)	1.34, 102.22	20–50^21^	19.18^*#^ (0.39)	0.35, 97.03	20–50^21^
Serum ferritin (μg/l)	104.12 (12.04)	1.00, 2877.17	12–150^21^	39.40^*#^ (5.10)	0.28, 2678.53	12–150^21^
Hb (g/dl)	13.30 (0.79)	4.70, 22.90	13–18^21^	12.00^*#^ (0.67)	4.70, 31.10	12–16^21^
BMI (kg/m^2^)	20.80 (0.15)	13.76, 65.39	18.5–24.9^20^	26.13* (0.21)	14.60, 53.64	18.5–24.9^20^
WC (cm)	73.95 (0.35)	53.60, 126.20	≤ 80^23^	77.25^*#^ (0.42)	46.50, 130.20	≤ 80^23^
WHR	0.84 (0.00)	0.59, 1.52	≤ 0.95^20^	0.76^*#^ (0.00)	0.47, 1.00	≤ 0.80^20^
Body fat (%)	20.63 (0.26)	9.17, 39.30		47.06^*#^ (0.45)	13.30, 83.79	
TSF (mm)	7.36 (0.19)	1.80, 37.30		18.89^*#^ (0.33)	3.50, 52.10	
SSF (mm)	9.63 (0.22)	3.00, 40.00		18.69^*#^ (0.42)	4.40, 60.00	

*Significant difference between men and women before adjusting for age, BMI and smoking (*p* < 0.05). ^#^Significant difference between men and women after adjusting for age, BMI and smoking (*p* < 0.05). BMI: body mass index, G-Mean: geometric mean, Hb: haemoglobin, SE: standard error, TIBC: total iron binding capacity, TSF: triceps skinfold, SSF: subscapular skinfold, WC: waist circumference, WHR: waist-to-hip ratio.

Correlations between iron indices and anthropometric indicators for both men and women are displayed in Table 2. In men, the strongest correlations were found between ferritin concentrations and WC (*rs* = 0.359, *p* < 0.01) and ferritin concentrations and WHR (*rs* = 0.396, *p* < 0.01). Likewise in women, the strongest correlations were found between ferritin concentrations and WC (*rs* = 0.232, *p* < 0.01) and ferritin concentrations and WHR (*rs* = 0.319, *p* < 0.01).

**Table 2:** Correlations Of Iron And Anthropometric Indices Of Participants

	*Iron Indices*									
*Anthropometric indices*	Serum Fe (μmol/l)		TIBC (μmol/l)		Transferrin saturation (%)		Ferritin (μg/l)		Hb (g/dl)	
	*(r_s_ )*	*(r_p_ )*	*(r_s_ )*	*(r_p_ )*	*(r_s_ )*	*(r_p_ )*	*(r_s_ )*	*(r_p_ )*	*(r_s_ )*	*(r_p_ )*
*Men*										
BMI (kg/m^2^)	0.015	–	119**	–	–0.032	–	0.141**	–	–0.056	–
WC (cm)	0.010	–0.043	0.000	0.004	0.006	–0.049	0.359**	0.068	–0.047	–0.064
WHR	0.027	0.067	–0.157**	–0.004	0.079*	0.062	396**	0.014	0.003	0.016
Body fat (%)	–0.048	–0.009	–0.029	0.008	–0.	0.012	0.308**	0.102	–0.111*	–0.096
TSF (mm)	0.012	–0.044	0.097*	0.051	0.071	–0.052	0.076*	–0.028	–0.022	–0.056
SSF (mm)	0.019	–0.031	0.035	0.001	–0.005	–0.027	0.141**	0.010	–0.018	–0.059
*Women*										
BMI (kg/m^2^)	–0.039	–	–0.041	–	–0.021	–	0.126**	–	–0.065*	–
WC (cm)	–0.036	–0.007	–0.087**	–0.045	–0.008	0.014	0.232**	0.024	–0.008	0.089
WHR	–0.044	–0.016	–0.166**	–0.026	0.004	0.008	0.319**	0.031	0.083*	0.126
Body fat (%)	–0.013	–0.021	–0.023	0.082	–0.002	–0.054	0.126**	0.012	–0.063	–0.011
TSF (mm)	–0.062	–0.053	–0.025	–0.012	–0.048	–0.047	0.040	–0.059	–0.037	–0.015
SSF (mm)	–0.029	–0.035	–0.040	–0.008	–0.014	–0.027	0.105**	–0.038	0.072*	–0.025

**Spearman correlation coefficient is significant at the 0.01 level (2-tailed). *Spearman correlation coefficient is significant at the 0.05 level (2-tailed). *r*_s_: Spearman correlation; *r*_p_: partial correlation after adjusting for age, BMI and smoking. BMI: body mass index, Hb: haemoglobin, SD: standard deviation, TIBC: total iron-binding capacity, TSF: triceps skinfold, SSF:, subscapular skinfold, WC: waist circumference, WHR: waist-to-hip ratio.

Iron indices are compared according to WHR and gender categories in Table 3. In men, mean (geometric) serum ferritin concentrations were significantly higher in the high-WHR group than the normal-WHR group (*p* < 0.0001), although this disappeared after adjusting for age, BMI and smoking. In addition, mean (geometric) serum iron concentration was also significantly higher in the high-WHR group than the normal-WHR group (*p* = 0.020) after adjusting for age, BMI and smoking.

**Table 3:** Comparison Of Iron Indices According To WHR Categories

	*Normal WHR*		*High WHR*	
*Variable*	*G-Mean (SE)*	*Min, max*	*G-Mean (SE)*	*Min, max*
*Men*	*(WHR > 0.95, n = 681, 95.78%)*		*(WHR > 0.95, n = 30, 4.22%)*	
Serum Fe (μmol/l)	16.43 (0.32)	0.74, 63.94	18.40^#^ (2.48)	4.73, 73.42
Serum TIBC (μmol/l)	63.94 (0.50)	28.77, 197.22	62.17 (2.53)	42.57, 102.42
Transferrin saturation (%)	25.71 (0.53)	1.34, 102.22	29.61 (3.26)	7.41, 79.95
Serum ferritin (μg/l)	100.80 (11.48)	1.00, 2877.17	217.28* (111.57)	30.00, 2427.20
Hb (g/dl)	13.29 (0.08)	4.70, 22.90	13.60 (0.51)	9.80, 21.10
*Women*	*(WHR > 0.80, n = 697, 73.21%)*		*(WHR > 0.80, n = 255, 26.79%)*	
Serum Fe (μmol/l)	13.26 (0.29)	1.18, 56.30	12.78 (0.45)	0.26, 59.85
Serum TIBC (μmol/l)	69.34 (0.52)	29.13, 171.12	65.21^*#^ (0.80)	36.93, 157.26
Transferrin saturation (%)	19.05 (0.45)	0.74, 85.46	19.54 (0.77)	0.35, 97.03
Serum ferritin (μg/l)	32.29 (5.44)	0.28, 2678.53	67.86^*#^ (11.44)	0.50, 1951.17
Hb (g/dl)	11.90 (0.07)	4.70, 20.50	12.27^*#^ (0.16)	5.70, 31.10

*Significant difference between normal and high WHR before adjusting for age, BMI and smoking (*p* < 0.05). ^#^Significant difference between normal and high WHR after adjusting for age, BMI and smoking (*p* < 0.05). G-Mean: geometric mean, Hb: haemoglobin, SE: standard error, TIBC: total iron-binding capacity, WHR: waist-to-hip ratio.

In women, mean (geometric) serum ferritin and haemoglobin concentrations were significantly higher in the high-WHR group than the normal-WHR group before (*p* < 0.0001, *p* = 0.003, respectively) and after (0.004, *p* = 0.018, respectively) adjusting for age, BMI and smoking. Women in the normal-WHR group had higher mean (geometric) serum TIBC than those in the high-WHR group before (*p* < 0.0001) and after (*p* = 0.019) adjusting for age, BMI and smoking. No significant differences were observed for serum iron concentration and transferrin saturation between the two WHR categories before and after adjusting for age, BMI and smoking.

The comparison of anthropometric indices according to three different ferritin concentrations (low, normal and high) is presented in Table 4. In men, mean (geometric) WC (*p* < 0.0001) and WHR (*p* < 0.0001) were significantly higher in the high-ferritin group than the low-ferritin group before adjusting for age, BMI and smoking but WC only remained significant (*p* = 0.014) after adjusting for age, BMI and smoking. Moreover, mean (geometric) WC (*p* < 0.0001) and WHR (*p* < 0.0001) of the men were significantly higher in the high-ferritin group than the normal-ferritin group before adjusting for age, BMI and smoking. This was not retained after adjusting for age, BMI and smoking. A significantly higher mean (geometric) BMI (*p* = 0.001) was found in the high-ferritin group compared to the normal-ferritin group before adjusting for age and smoking. The high-ferritin group had a significantly higher mean (geometric) body fat compared to the low-ferritin group (*p* = 0.015) and the normal-ferritin group (*p* < 0.0001) before adjusting for age, BMI and smoking.

**Table 4:** Comparison Of Anthropometric Indices According To Serum Ferritin Concentration

	*Low-ferritin group (ferritin < 12 μg/l)*		*Normal-ferritin group (ferritin 12–150 μg/l)*		*High-ferritin group (ferritin < 150 μg/l)*	
*Variable*	*G-Mean (SE)*	*Min, max*	*G-Mean (SE)*	*Min, max*	*G-Mean (SE)*	*Min, max*
*Men*	*(n = 23, 3.23%)*		*(n = 418, 58.79%)*		*(n = 270, 37.97%)*	
BMI (kg/m^2^)	20.64 (0.73)	16.42, 31.50	20.29 (0.15)	14.71, 36.91	21.64^®^ (0.31)	13.76, 65.39
WC (cm)	69.08 (1.30)	60.20, 83.40	72.00^#^ (0.40)	53.60, 114.50	77.52^+®$^ (0.61)	57.00, 126.20
WHR	0.80 (0.01)	0.71, 0.92	0.82* (0.00)	0.59, 1.05	0.86^+®^ (0.00)	0.71, 1.52
Body fat (%)	19.29 (1.48)	12.53, 36.71	19.97 (0.29)	9.22, 37.15	22.29^+®^ (0.52)	9.17, 39.30
TSF (mm)	7.75 (0.85)	4.90, 18.20	7.09 (0.24)	2.00, 37.30	7.77 (0.32)	1.80, 37.10
SSF (mm)	8.69 (1.01)	5.50, 19.90	9.27 (0.27)	3.40, 40.00	10.32 (0.40)	3.00, 38.10
*Women*	*(n = 155, 16.28%)*		*(n = 662, 69.54%)*		*(n = 135, 14.18%)*	
BMI (kg/m^2^)	24.50 (0.50)	15.45, 45.96	26.40* (0.26)	15.39, 53.64	26.74 (0.64)	14.60, 48.76
WC (cm)	72.47 (0.89)	46.50, 107.50	77.50* (0.50)	54.50, 130.20	81.83^+®$@^ (1.15)	55.00, 119.60
WHR	0.73 (0.00)	0.57, 0.96	0.75* (0.00)	0.47, 1.00	0.80^+®$@^ (0.00)	0.64, 0.99
Body fat (%)	45.01 (0.87)	28.17, 68.40	47.81 (0.57)	13.30, 83.79	47.33 (1.42)	28.79, 70.55
TSF (mm)	17.86 (0.80)	4.40, 51.70	19.10 (0.38)	4.40, 50.20	19.12 (1.06)	3.50, 52.10
SSF (mm)	17.15 (0.92)	5.40, 52.90	18.75 (0.51)	4.60, 56.60	20.30 (1.23)	4.40, 60.00

*Significant difference between negative and normal iron balance before adjusting for age, BMI and smoking at *p* < 0.05. ^+^Significant difference between negative and positive iron balance before adjusting for age, BMI and smoking at *p* < 0.05. ^®^Significant difference between normal and positive before adjusting for age, BMI and smoking at *p* < 0.05. ^#^Significant difference between negative and normal iron balance after adjusting for age, BMI and smoking at *p* < 0.05. ^$^Significant difference between negative and positive iron balance group after adjusting for age, BMI and smoking at *p* < 0.05. ^@^Significant difference between normal and positive iron balance after adjusting for age, BMI and smoking at *p* < 0.05. BMI: body mass index, G-Mean: geometric mean, SE: standard error, SSF: subscapular skinfold, TSF: triceps skinfold, WC: waist circumference, WHR: waist-to-hip ratio.

For women, mean (geometric) WC and WHR were significantly higher in the high-ferritin group than the low-ferritin group before (*p* < 0.0001, *p* < 0.0001, respectively) and after (*p* = 0.002, *p* = 0.018, respectively) adjusting for age, BMI and smoking. WC and WHR were also higher in the high-ferritin group than the normal-ferritin group before (*p* = 0.033, *p* = 0.005, respectively) and after (*p* < 0.0001, *p* = 0.014, respectively) adjusting for age, BMI and smoking. Women in the normal-ferritin group had higher mean (geometric) BMI (*p* = 0.032) than those in the low-ferritin group before adjusting for age and smoking.

Table 5 compares the mean (geometric) iron indices among four BMI categories. In men, mean (geometric) serum TIBC was significantly higher in the normal-weight group compared to the underweight group before (*p* = 0.016) and after (*p* = 0.018) adjusting for age and smoking. Additionally, mean (geometric) serum TIBC was significantly higher in the overweight than the underweight (*p* = 0.047) groups after controlling for age and smoking. No significant differences were observed in ferritin, serum iron and haemoglobin concentrations and percentage transferrin saturation among men in the different BMI categories before and after adjusting for age and smoking.

**Table 5:** Comparison Of Iron Indices According To BMI Categories

	*Underweight (BMI < 18.50 kg/m^2^)*		*Normal weight (BMI 18.50–24.90 kg/m^2^)*		*Overweight (BMI 25.00–29.90 kg/m^2^)*		*Obese (BMI^3^ ≥ 30.00 kg/m^2^)*	
*Variable*	*G-Mean (SE)*	*Min, max*	*G-Mean (SE)*	*Min, max*	*G-Mean (SE)*	*Min, max*	*G-Mean (SE)*	*Min, max*
*Men*	*(n = 165, 23.21%)*		*(n = 451, 63.43%)*		*(n = 69, 9.70%)*		*(n = 26, 3.66%)*	
Serum Fe (μmol/l)	16.05 (0.77)	0.74, 73.42	16.75 (0.40)	1.02, 63.94	16.46 (0.91)	3.25, 38.04	15.67 (1.40)	3.62, 36.57
Serum TIBC (μmol/l)	61.62 (0.94)	28.77, 97.77	64.49^*#^ (0.65)	36.54, 197.22	64.84^$^ (1.55)	41.64, 109.50	64.89 (15.67)	46.26, 84.48
Transferrin saturation (%)	26.05 (1.28)	2.20, 97.36	25.97 (0.65)	1.34, 102.22	25.38 (1.37)	5.02, 63.14	24.15 (2.12)	4.57, 54.91
Serum ferritin (μg/l)	92.26 (20.90)	2.50, 2387.60	101.59 (16.95)	1.00, 2877.17	134.52 (16.81)	1.54, 807.22	174.02 (46.58)	5.86, 956.88
Hb (g/dl)	13.43 (0.18)	7.40, 22.90	13.22 (0.09)	4.70, 21.10	13.25 (0.21)	8.90, 17.70	13.90 (0.47)	11.00, 21.10
*Women*	*(n = 56, 5.90%)*		*(n = 380, 40.049%)*		*(n = 242, 25.50%)*		*(n = 271, 28.56%)*	
Serum Fe (μmol/l)	11.75 (1.36)	1.85, 51.55	13.12 (0.39)	1.18, 40.96	14.33 (0.46)	2.30, 56.30	12.42^μ♀σ ^(0.42)	0.26, 59.85
Serum TIBC (μmol/l)	68.95 (2.83)	29.13, 171.12	68.49 (0.69)	32.97, 120.96	68.46 (0.81)	39.33, 122.82	67.51 (0.82)	36.93, 157.26
Transferrin saturation (%)	17.04 (2.19)	1.30, 83.96	19.03 (0.60)	0.74, 64.79	20.92 (0.77)	2.35, 85.46	18.35^μσ^ (0.69)	0.35, 97.03
Serum ferritin (μg/l)	46.69 (39.16)	1.24, 1951.17	31.60^*#^ (6.45)	0.28, 1470.70	43.24^+$^ (12.66)	0.83, 2678.53	47.16^®@^ (6.60)	0.50, 775.58
Hb (g/dl)	12.43 (0.46)	6.67, 31.10	12.05* (0.98)	6.10, 20.90	12.00^+^ (0.12)	4.70, 19.80	11.87^®@^ (0.12)	5.70, 19.60

*Significant difference between underweight and normal weight before adjusting for age and smoking (*p* < 0.05). ^+^Significant difference between underweight and pre-obese before adjusting for age and smoking (*p* < 0.05). ^®^Significant difference between underweight and obese before adjusting for age and smoking (*p* < 0.05). ^♀^Significant difference between normal weight and obese before adjusting for age and smoking (*p* < 0.05). ^μ^Significant difference between pre-obese and obese before adjusting for age and smoking (*p* < 0.05). ^#^Significant difference between underweight and normal weight after adjusting for age and smoking (*p* < 0.05). ^$^Significant difference between underweight and pre-obese after adjusting for age and smoking (*p* < 0.05). ^@^Significant difference between underweight and obese after adjusting for age and smoking (*p* < 0.05). ^σ^Significant difference between pre-obese and obese after adjusting for age and smoking (*p* < 0.05). BMI: body mass index, G-Mean: geometric mean, Hb: haemoglobin, SE: standard error, TIBC: total iron-binding capacity.

For women, mean (geometric) serum ferritin concentration was significantly higher in the underweight group than the normal-weight, overweight and obese groups before (*p* = 0.001, *p* = 0.020, *p* = 0.014, respectively) and after (*p* = 0.017, *p* = 0.037, *p* = 0.007, respectively) controlling for age and smoking. A significantly higher mean (geometric) haemoglobin concentration was observed in the underweight compared to the overweight (*p* = 0.036) and obese (*p* = 0.013) groups, although only the difference between the underweight and obese was retained (*p* = 0.022) after adjusting for age and smoking.

Percentage transferrin saturation was higher in the overweight than obese women before (*p* = 0.014) and after (*p* = 0.016) adjusting for age and smoking. Obese women had a lower mean (geometric) serum iron than the normal-weight (*p* = 0.047) and overweight (*p* = 0.006) women but only the difference between the overweight and obese remained significant (*p* = 0.013) after adjusting for age and smoking. No significant differences in TIBC were observed for the different BMI categories before and after adjusting for age and smoking.

## Discussion

This is the first study to our knowledge that assessed the relationship between iron indices and anthropometric CVD markers in an African population. This study employed anthropometric measures, which have been reported to be associated with CVD risk. These factors include WC, WHR, triceps and subscapular skinfold thicknesses (TSF and SSF), percentage body fat and BMI.^15^,^24^ The results showed that both men and women in the high-WHR category had higher ferritin concentrations than those in the normal-WHR category. A positive association between ferritin concentrations and BMI, WC, WHR, percentage body fat and SSF was demonstrated for both men and women; although this disappeared after adjusting for age, BMI and smoking. WC and WHR increased with increasing ferritin concentrations in both men and women. Serum iron concentrations decreased with increasing BMI in women only.

The results obtained in this study are similar to the work of Gillum^25^ who reported that serum ferritin concentrations associated positively with WHR and BMI in Mexican-American men. Norwegian men aged 20–49 years were also reported to have a mean serum ferritin concentration that related positively to their mean BMI.^26^ Additionally, a study conducted on diabetic patients reported that serum ferritin concentrations correlated positively with visceral fat and subcutaneous fat area. This study excluded patients with high C-reactive protein concentrations in order to exclude elevation of ferritin that may have been caused by inflammation. The authors therefore concluded that ferritin concentrations might be a useful indicator of systemic percentage fat.^27^

Conversely, Eftekhari *et al*.^28^ reported a negative correlation between serum ferritin concentrations and BMI in adolescent Iranian girls. This is in contrast to the results of this study that showed a positive correlation between serum ferritin concentrations and BMI. The authors attributed the result to the age group of the study population. Adolescence, being a peculiar stage of growth, is characterised by a growth spurt and increased iron requirements. The onset of menstruation in girls makes their iron need greater than that of boys.^29^ The present study consisted of men and women across all age groups, which makes it less comparable to the Iranian study. Studies have also reported appreciable differences in the prevalence of obesity among different ethnic groups.^30^,^31^ Chambers *et al*.^32^ reported an inverse relationship between body fat distribution and serum iron concentrations in Hispanic women but not in white, African-American or Asian women.

In the present study, a lower serum iron concentration in the obese than the normal-weight and pre-obese women was observed. Serum iron concentrations in the obese remained significantly lower than in the pre-obese women after adjusting for age and smoking. This is congruent with the findings by Tussing-Humphreys,^33^ who reported that the prevalence of iron deficiency was higher in overweight girls. Moreover, serum iron concentration was reported to decrease as BMI increased in adolescent Iranian girls.^27^ The National Health and Nutrition Examination Survey I^34^ reported that higher BMI was significantly associated with lower serum iron concentrations in women. Serum iron concentration is, however, not a sensitive marker of iron status, due to diurnal changes.^35^

An understanding of the link between fat deposition and ferritin secretion may offer an explanation of some of these observations. During fat deposition, lipid biosynthesis increases, which might lead to an increase in iron-induced lipid oxidation as a result of the reactivity of intracellular iron with lipids. There is a probability that ferritin levels are elevated to act as an iron cytoprotective agent.^36^ Therefore, increased ferritin concentrations may be an adaptive mechanism to reduce iron-induced oxidative stress, which could explain the positive correlation between ferritin concentrations and anthropometric CVD risk factors.

Ferritin is an acute-phase reactant, which increases in concentration during inflammation. Since obesity is considered an inflammatory state,^37^ it could serve as an additional explanation for the positive association between ferritin concentrations and anthropometric indicators. However, serum ferritin concentrations can be influenced by other inflammatory conditions resulting from infection. A deficient iron store owing to greater iron requirements in obese adults because of their larger blood volume^38^ has also been proposed to be the mechanism involved in the iron deficiency–obesity association. Functional iron deficiency can occur during inflammation (even when iron stores are optimal) as a result of impairment of the normal physiological systems for transport of iron to the target tissue.^10^

It is not clear which precedes the other, obesity or iron deficiency. Iron takes part in diverse physiological functions such as transport of oxygen by haemoglobin and myoglobin, energy metabolism by the haeme-containing proteins of the mitochondrial electron transport apparatus and the conversion of ribose to deoxyribose nucleic acids by the iron-containing ribonucleotide reductase, which is required for the propagation of genetic information.^10^ Given the critical dependence of body tissues on iron, it is possible that its deficiency could result in accumulation of fat in the body tissues. Impaired fat oxidation has been reported to be a risk factor for excess weight gain in several populations known to be susceptible to obesity.^39^,^40^ Fat that is not oxidised must be stored, which can result in increased fat deposition with time. Iron deficiency has also been linked to problems with work and exercise capacity among adults,^41^ which may eventually lead to a sedentary lifestyle and weight gain.

Lipid deposition allows efficient storage of maximal calories in adipose tissue located beneath the skin (subcutaneous fat), around internal organs (visceral fat) and in the yellow bone marrow.^42^ Subcutaneous fat tissue stores about 80% of all body fat, and excess fat is stored in other storage tissues, such as the intra-abdominal tissue when the subcutaneous tissue reaches a threshold level.^43^ Therefore, storage of excess fat in the central region may be a signal for abnormal fat storage, which has adverse health implications. It has been found that body fat distribution, especially abdominal fat is more important than total body fat in the aetiology of CVD.^44^ William *et al*.^45^ identified measurements at or above the waist to be most associated with disease risk for both genders. However, in the present study, 79% of men and 60% of women were within the optimal cut-off point.^24^

There is speculation that hepcidin, a peptide hormone involved in the regulation of intracellular iron, may be involved in the association of obesity and iron deficiency.^46^ Hepcidin is an acute-phase reactant that is stimulated in inflammation, including obesity.^47^ Moreover, recent discoveries indicated that adipocytes are not just passive organs for fat storage, instead, they are also endocrine organs which play regulatory roles in whole-body physiology.^48^ Increased secretion of hepcidin has been reported to inhibit the release of non-haeme iron from macrophages.^49^ It is possible that increased expression of hepcidin in obese individuals interferes with iron absorption, thereby resulting in iron deficiency. Unfortunately, hepcidin was not measured in the present study as it might have helped to clarify the situation.

Gender has been reported to be one of the factors that influence serum ferritin concentrations.^50^ A significant difference in serum ferritin concentrations was demonstrated between men and women in this study. Leggett *et al*.^51^ found that the pattern of ferritin increase varied in men and women. Ferritin concentrations in women, although increasing, remained low until after menopause, whereas those of men continued to increase, reaching a peak in their fourth decade, when iron stores are expected to be stable following the growth period. This suggests that the observed difference between men and women may be as a result of physiological differences (i.e. menstruation and hormone secretion) that affect iron storage. Moreover, the pattern of fat distribution in men has been reported to differ from that in women. Women usually show greater lower body fat distribution (gynoid) while men show more upper body fat distribution (android).^42^ The present study supports these findings as men had significantly higher WC than women.

This study illustrates conclusively that a relationship exists between some measures of iron status and certain anthropometric CVD risk markers, particularly ferritin concentrations and WC or WHR. This implies that individuals who fell within the positive iron-balance group, the at-risk group for iron overload, may additionally be at a greater risk of developing CVD in this particular population. However, due to the nature of the design of this study, causality cannot be established.

## Implication for health and research

Iron deficiency is the most common micronutrient deficiency in the world^2^ and it has been a priority for most countries when addressing issues on health. South Africa is among the countries with moderate iron deficiency.^52^ The Department of Health in South Africa is using an adapted form of UNICEF’s conceptual framework to address malnutrition, which means action is being taken at all levels of causation.^53^ Strategies employed include provision of iron supplements to pregnant and postpartum women, fortification of several food vehicles and dietary diversification (using the food-based dietary guidelines). It has been reported that some indicators are improving while others are worsening over the years. In addition, reports have shown that nutritional status varies considerably among the nine provinces and possibly within each province.^54^,^55^

On the other hand, iron overload has increasingly been recognised as a public health issue in African populations, particularly southern Africa, where the consumption of traditional beer has been identified as a major contributor.^10^ However, the results of the present study suggest that increased abdominal obesity may be another major contributor to increased iron stores in this population. Therefore, it may be pertinent to scale up interventions to reduce obesity in this population, alongside ongoing iron-intervention programmes. Furthermore, consideration should be given to the use of different and locally relevant strategies for different provinces, or possibly different municipalities within a province. To further enhance the effort put into improving health and nutrition, different and relevant strategies addressing the various public health concerns of our population would be helpful in arriving at the desired nutritional and health goals.

## References

[1] Vorster HH (2002). The emergence of cardiovascular disease during urbanisation of Africans.. Publ Hlth Nutr.

[2] (2008). Worldwide prevalence of anaemia 1993–2005.. WHO Global Database on Anaemia.

[3] Kurniawan YA, Muslimatun S, Achadi EL, Sastroamidjojo S (2006). Anaemia and iron deficiency anaemia among young adolescents girls from the peri urban coastal area of Indonesia.. Asia Pac J Clin Nutr.

[4] Tolentino K, Friedman JF (2007). An update on anemia in less developed countries.. Am J Trop Med Hyg.

[5] Mamabolo RL, Albets M, Steyn NP, Delemarre-Van De Wall HA, Nthangeli NG, Levitt NS (2004). Evaluation of the effectiveness of iron and folate supplementation during pregnancy in a rural area of Limpopo Province.. S Afr J Clin Nutr.

[6] Dannhauser A, Bam R, Joubert G (1999). Iron status of pregnant women attending the antenatal clinic at Pelonomi Hospital, Bloemfontein.. S Afr J Clin Nutr.

[7] Kruger M, Dhansay MA, Van Staden E, Faber M, Badenhorst CJ, Mansvelt EPG (1994). Anaemia and iron deficiency in women in the third trimester of pregnancy receiving selective iron supplementation.. S Afr J Food Sci Nutr.

[8] Bopape MM, Mbhenyane XG, Alberts M (2008). The prevalence of anaemia and selected micronutrient status in pregnant teenagers of Polokwane municipality in the Limpopo Province.. S Afr J Clin Nutri.

[9] Charlton KE, Kruger M, Labadarios D, Wolmarans P, Aronson I (1997). Iron, folate and vitamin B_12_ status of an elderly South African population.. Eur J Clin Nutr.

[10] Kew MC, Asare GA (2007). Dietary iron overload in the African and hepatocellular carcinoma.. Liver Int.

[11] Vorster HH, Bourne LT, Venter CS, Oosthuizen W (1999). Contribution of nutrition to the health transition in developing countries: Framework for research and intervention.. Nutr Rev.

[12] (2007). Report of the 2003 South African demographic and health survey.. www.sahealthinfo.org/lifestyle/adult.pdf..

[13] Robinson LE, Graham TE (2004). Metabolic syndrome, a cardiovascular disease risk factor: Role of adipocytokines and impact of diet and physical activity.. J Appl Physiol.

[14] Williams MJ, Poulton R, William S (2002). Relationship of serum ferritin with cardiovascular risk factors and inflammation in young men and women.. Atherosclerosis.

[15] Schulze MB, Christian H, Manuela M, Berg M, Kurt H, Heiner B (200). Comparison of anthropometric characteristics in predicting the incidence of type 2 diabetes in the EPIC-Potsdam study.. Diabetes Care.

[16] Vorster HH, Wissing MP, Venter CS, Kruger HS, Kruger A, Malan NT (2000). The impact of urbanization on physical and mental health of Africans in the Northwest province of South Africa: The THUSA study.. S Afri J Sci.

[17] Norton K, Olds T (1996). Anthropometrica.. A Textbook of Body Measurement for Sports and Health Courses..

[18] Siri WE, Tobias CA, Lawrence JH (1956). The gross composition of the body.. Advances in Bbiological and Medical Physics..

[19] Durnin JVGA, Womersley J (1974). Body fat assessed from total body density and its estimation from skinfold.. Br J Nutr.

[20] (1997). Obesity: Preventing and managing the global epidemic. Report of WHO consultation on obesity..

[21] Kasdan TS, Malian LK, Escott, Stump A (2000). Medical Nutrition therapy for anaemia.. Krause’s Food, Nutrition and Diet Therapy..

[22] Cook JD, Finch CA (1979). Assessing iron status of a population.. Am J Clin Nutr.

[23] Beneke J, Ridder JH, Underhay C, Kruger A, Van Rooyen JM, Ellis S (2009). Optimal waist circumference cut-off values for abdominal obesity in black South African men and women.. S Afri J Sports Med.

[24] Mueller WH, Wear ML, Hanis CL, Emerson JB, Barton SA, Hewett-Emmett D, Schull WJ (1991). Which measure of body fat distribution is best for epidemiologic research?. Am J Epidemiol.

[25] Gillum RF (2001). Association of serum ferritin and indices of body fat distribution and obesity in Mexican American men: the Third National Health and Nutrition Examination Survey.. Int J Obes Relat Metab Disord.

[26] Oshuag A, Bugge KH, Bjonnes CH, Borch-Johnsen B, Neslein IL (1995). Associations between serum ferritin and cardiovascular risk factors in healthy young men. A cross-sectional study.. Eur J Clin Nutr.

[27] Iwasaki T, Nakajima A, Yoneda m, Yamada y, Mukasa K, Fujita K (2005). Serum ferritin is associated with visceral fat area and subcutaneous fat area.. Diabetes Care.

[28] Eftekhari MH, Mozaffari-KhosravI H, Shidfar F (2009). The relationship between BMI and iron status in iron deficient adolescent Iranian girls.. Publ Hlth Nutr.

[29] Dallman PR (1986). Biochemical basis for the manifestation of iron deficiency.. Ann Rev Nutr.

[30] Hedley AA, Ogden CL, Johnson CL, Carroll MD, Curtin LR, Flegal KM (2004). Prevalence of overweight and obesity among US children, adolescents, and adults, 1999–2002.. J Am Med Assoc.

[31] Flegal KM, Carrol MD, Kuczmarski RJ, Johnson CL (1998). Overweight and trends, 1960–1994.. Int J Obes Relat Metab Disord.

[32] Chambers EC, Heshka S, Gallagher D, Wang J, Pi-Sunyer X, Pierson RN (2006). Serum iron and body fat distribution in a multiethnic cohort of adults living in New York.. J Am Dietetic Assoc.

[33] Tussing-Humphreys LM, Liang H, Nemeth E, Freels S, Braunschweig CA (2009). Excess adiposity, inflammation, and iron-deficiency in female adolescents.. J Am Diet Assoc.

[34] Micozzi MS, Albanes D, Stevens RG (1989). Relation of body size and composition to clinical biochemical and hematologic indices in US men and women.. Am J Clin Nutr.

[35] Bothwell TH, Charlton RW, Cook JD, Finch CA (1979). Iron Metabolism in Man..

[36] Festa M, Ricciardelli G, Mele G, Pietropaolo C, Ruffo A (2000). Overexpression of H ferritin and upregulation of iron regulatory protein genes during differentiation of 3T3–L1 pre-adipocytes.. J Biol Chem.

[37] Yanoff LB, Serbring NG, Mchugh T, Remaley AT, Yanovski JA (2007). Inflammation and iron deficiency in the hypoferrimia of obesity.. Int J Obes.

[38] Failla ML, Kennedy ML, Chen ML (1988). Iron metabolism in genetically obese (ob/ob) mice.. J Nutr.

[39] Astrup A, Buemann B, Christensen N, Toubro S (1994). Failure to increase lipid oxidation in response to increasing dietary fat content in formerly obese women.. Am J Physiol.

[40] Zurlo F, Lillioka S, Esposito-del Puente A (1990). Low ratio of fat to carbohydrate oxidation as predictor of weight gain: of 24-h RQ.. Am J Physiol.

[41] Hass JD, Brownlie T (2001). Iron deficiency and reduced work capacity: a critical review of the research to determine a causal relationship.. J Nutr.

[42] Weiss R (2007). Fat distribution and storage: how much, where, and how?. Eur J Endocrinol.

[43] Arner P (1997). Regional adiposity in man.. J Endocrinol.

[44] Märin P, Andersson B, Ottosson M, Olbe L, Chowdhury B, Kvist H (1992). The morphology and metabolism of intra-abdominal adipose tissue in men.. Metabolism.

[45] William HM, Mary LW, Craig LH, James BE, Sara AB, Hewett-Emmett D, William JS (1991). Which measure of body fat distribution is best for epidemiologic research?. Am J Epidemiol.

[46] McClung JP, Karl JP (2008). Iron deficiency and obesity: the contribution of inflammation and diminished iron absorption.. Nutr Rev.

[47] Nemeth E, Valore EV, Territo M, Schiller G, Lichtenstein A, Ganz T (2003). Hepcidin, a putative mediator of anemia of inflammation, is a type II acute-phase protein.. Blood.

[48] Andrew S, Greenber G, Martin SO (2006). Obesity and the role of adipose tissue in inflammation and metabolism.. Am J Clin Nutri.

[49] Knutson MD, Oukka M, Koss LM, Aydemir F, Wessling-Resnick M (2005). Iron release from macrophages after erythrophagocytosis is up-regulated by ferroportin 1 overexpression and down-regulated by hepcidin.. Proc Natl Acad Sci USA.

[50] Watter BO, Miller FM, Worwood M (1973). Ferritn concentration and iron stores in normal subjects.. J Clin Pathol.

[51] Leggett BA, Brown NN, Bryant SJ, Duplock L, Powell LW, Halliday JW (1990). Factors affecting the concentrtions of ferritin in serum in a healthy Australian population.. Clin Chem.

[52] (2001). Iron deficiency anaemia: assessment, prevention, and control. A guide for programme managers..

[53] (1998). Integrated Nutrition Programme for South Africa – Broad guidelines for implementation..

[54] Faber M, Wenhold F (2007). Nutrition in contemporary South Africa.. Water SA.

[55] Vorster HH, Oosthuizen W, Jerling JC, Veldman FJ, Burger HM (1997). The Nutritional status of South Africans. A review of the literature from 1975–1996.. Hlth Syst Trust.

[56] Kruger HS, Kruger A, Vorster HH, Jooste PL, Wolmarans P (2005). Urbanisation of Africans in the North West province is associated with better micronutrient status: The Transition and health during Urbanisation study in South Africa.. Nutr Res.

